# Comparative metabolomics combined with genome sequencing provides insights into novel wolfberry-specific metabolites and their formation mechanisms

**DOI:** 10.3389/fpls.2024.1392175

**Published:** 2024-04-26

**Authors:** Qiyuan Long, Changjian Zhang, Hui Zhu, Yutong Zhou, Shuo Liu, Yanchen Liu, Xuemin Ma, Wei An, Jun Zhou, Jianhua Zhao, Yuanyuan Zhang, Cheng Jin

**Affiliations:** ^1^ School of Breeding and Multiplication (Sanya Institute of Breeding and Multiplication), Hainan University, Sanya, China; ^2^ School of Tropical Agriculture and Forestry, Hainan University, Haikou, Hainan, China; ^3^ Department of Forest Genetics and Plant Physiology, Swedish University of Agricultural Sciences, Umeå, Sweden; ^4^ National Wolfberry Engineering Research Center, Wolfberry Science Research Institute, Ningxia Academy of Agriculture and Forestry Sciences, Yinchuan, China; ^5^ College of Biological Science and Engineering, North Minzu University, Yinchuan, China

**Keywords:** metabolome, nutrition, riboflavin, phenyllactate, copy number variation

## Abstract

Wolfberry (*Lycium*, of the family Solanaceae) has special nutritional benefits due to its valuable metabolites. Here, 16 wolfberry-specific metabolites were identified by comparing the metabolome of wolfberry with those of six species, including maize, rice, wheat, soybean, tomato and grape. The copy numbers of the riboflavin and phenyllactate degradation genes *riboflavin kinase* (*RFK*) and *phenyllactate UDP-glycosyltransferase* (*UGT1*) were lower in wolfberry than in other species, while the copy number of the phenyllactate synthesis gene *hydroxyphenyl-pyruvate reductase* (*HPPR*) was higher in wolfberry, suggesting that the copy number variation of these genes among species may be the main reason for the specific accumulation of riboflavin and phenyllactate in wolfberry. Moreover, the metabolome-based neighbor-joining tree revealed distinct clustering of monocots and dicots, suggesting that metabolites could reflect the evolutionary relationship among those species. Taken together, we identified 16 specific metabolites in wolfberry and provided new insight into the accumulation mechanism of species-specific metabolites at the genomic level.

## 1 Introduction

Wolfberry (*Lycium*, of the family Solanaceae) has excellent nutritional value, with a history dating back thousands of years (Jin et al., 2013). The wolfberry genus contains ~80 species, with a discrete geographic distribution from South and North America to Australia, Eurasia, the Pacific Islands, and South Africa. There are 7 species and 3 varieties in China, mainly distributed in the north (Levin and Miller, 2005). Consuming wolfberry could promote human health by nourishing the liver and kidneys, enhancing vision and regulating the immune system (Vidović et al., 2022). As a result, a large number of studies have assessed the nutritional value of wolfberry from various perspectives. For example, one of the key components responsible for the antioxidant, immunomodulatory, and anticancer effects in *Lycium barbarum* L. is *Lycium barbarum* polysaccharide (LBP) (Jin et al., 2013; Masci et al., 2018). *Lycium barbarum* fruits (LBFs) flavonoids are involved in prominent antioxidant, hypolipidemic, hypoglycemic, immunity-enhancing, and antitumor activities (Yang et al., 2022). The anthocyanins from *Lycium ruthenicum* Murray have a positive role in maintaining intestinal health and play an antioxidant role (Yan et al., 2018). Ascorbic acid and its derivatives in LBFs can regulate the intestinal flora in mice (Huang et al., 2020). However, except for these known active substances, wolfberry-specific metabolites are still poorly known.

To date, various metabolic methods have been applied to the determination of metabolites in wolfberry. Fifty-six volatile compounds in Ningxia goji berries were characterized by gas chromatography-spectrometry (GC-MS) and identified by gas chromatography-olfactometry (GC-O) and aroma dilution analysis (AEDA) (Lu et al., 2017). Using ultrahigh-performance liquid chromatography-quadrupole time-of-flight mass spectrometry (UPLC-Q-TOF/MS), 41 spermidine derivatives were tentatively characterized from LBFs (Ahad et al., 2020). Nine alkaloids were yielded in LBFs by spectroscopic analyses and chemical methods (Chen et al., 2021). Thirteen flavonoid compounds were identified in LBFs using LC-MS (Yang et al., 2022). Based on these metabolic methods, the comparative metabolome of different varieties (Zhang et al., 2016), tissues (Xiao et al., 2021) or development stages of wolfberry (Zhao et al., 2015) under various environmental conditions (Poggioni et al., 2022) has been extensively studied. For example, by comparing the metabolic groups of *Lycium barbarum*, *Lycium chinense*, and *Solanum lycopersicum*, the metabolic markers distinguishing *Lycium* and *Solanum* fruits were revealed (Dumont et al., 2020). However, there is still a large gap in metabolome comparison between wolfberry and different species, which needs further exploration.

To study the metabolic mechanism of wolfberry-specific substances, many studies have been carried out on the regulatory mechanism of metabolites during fruit ripening. The *LbNCED1* transcript level was transcriptionally activated by the developmental cues of *Lycium* fruit, enhancing the accumulation of abscisic acid (ABA), thereby promoting anthocyanin production and leading to fruit coloration (Li et al., 2019). Distinction in the expression patterns of 22 transcription regulators may be the main reason for the morphological and phytochemical differences between *L. barbarum* (LB) and *L. ruthenicum* (LR) fruits at five developmental stages (Zhao et al., 2020b). Recent research has shown that *LbNR* (nitric reductase (NR) from *L. barbarum*) inhibited anthocyanin biosynthesis and enhanced proanthocyanidin (PA) accumulation by regulating nitric oxide (NO) (Li et al., 2020a). Moreover, based on metabolome and transcriptome analysis, many key genes involved in metabolite synthesis were identified in wolfberry. For example, candidate genes for flavonoid biosynthesis were identified by conducting transcriptome and flavonoid metabolic profiling, and the molecular regulatory mechanism of *LrAN1b* on anthocyanins and fruit color was verified, which provided a new understanding of the potential mechanism of action of flavonoids (Li et al., 2020b). Through competitive transcriptome analysis between LB and LR, 38 MYB transcription factors that may regulate the fruit development of wolfberry were identified (Wang et al., 2020). Recently, the first reference genome of wolfberry was published (Cao et al., 2021), indicating that the genetic basis of metabolites in wolfberry can be analyzed at the genomic level.

To comprehensively explore the specific metabolites and their causes in wolfberry, we compared the metabolome of wolfberry, rice, maize, wheat, soybean, grape, and tomato. A total of 1043 distinct metabolic features were detected and were divided into 10 categories, 16 of which were identified as wolfberry-specific metabolites. Our results showed that metabolites could reflect the evolutionary relationship among different species. We further showed that the copy numbers of *RFK*, *HPPR*, and *UGT1* may be the main reasons for the specific accumulation of riboflavin and phenyllactate in wolfberry.

## 2 Materials and methods

### 2.1 Plant materials

To study the differences in metabolites between wolfberry (*Lycium*, of the family Solanaceae) and other species, we selected three monocot crops and three dicot crops as reference objects. The monocot crops included three major staple crops maize (*Zea mays* L.), rice (*Oryza sativa* L.) and wheat (*Triticum aestivum* L.), with rice being the model plant for monocots. The dicot crops included legume crop soybean (*Glycine max* (L.) Merr.) and the fruit crops tomato (*Solanum lycopersicum* L.) and grape (*Vitis vinifera* L.). Wolfberry (*L.barbarum* ‘Ningqi No.1’, *L. barbarum* var. *auranticarpum*, *L. ruthenicum*) was obtained from Yinchuan, Ningxia (E113°42′, N34°48′). Maize (Waxy maize, Red Waxy maize, Fruit maize, Sweet maize), Grape (Red grape, Green grape, Seedless red grape, Jufeng grape), Tomato (Pink tomato, Tomato, Cherry tomato, Millennium cherry tomato) were obtained from Haikou, Hainan (E110°20′, N20°02′). Soybeans (GDC058, GDC062, GDC063) were obtained from Zhengzhou, Henan (E113°42′, N34°48′). Wheat (Lumai 21, Heng 7228, Linmai No. 2, Xinong 529) was obtained from Zhaoxian, Hebei (E114°28′, N38°02′). Rice (Huanghuazhan, Nipponbare, Mimghui 63, Zhengshan 97, Zhonghua 11) was obtained from Hainan University (E110°20′, N20°02′).

### 2.2 Sample preparation and extraction

The samples were put in a lyophilizer for vacuum freeze-drying, and they were crushed in a mixer mill (MM 400; Retsch, Haan, Germany) for 1 min at 30 Hz. Next, 80 mg of the powdered sample was weighed into a 2 mL centrifuge tube, and 70% aqueous methanol (v/v) with lidocaine internal standard was added to extract the water-soluble metabolites (pure methanol was used to extract the fat-soluble metabolites). Then, the tube was vortexed for 10 seconds, allowed to stand for 10 min, repeated three times, and placed in a refrigerator at 4°C for 10-12 hours. Then, the sample was centrifuged (4°C, 10000 rpm, 10 min), the supernatant was pipetted, the water-soluble and fat-soluble metabolites were mixed 1:1 and filtered by a microporous filtration membrane (SCAA-104, 13 mm, 0.22 μm, Shanghai Anpu Experimental Technology Co., Ltd., http://www.anpel.com.cn/), and the sample was filtered into an injection bottle for storage for UPLC-MS analysis.

### 2.3 Detection of metabolites

The instruments used for LC-MS/MS analysis included UPLC-Q Exactive Plus Orbitrap HRMS and UPLC-Q-Trap 6500+ MS. The analytical column used was a C18 column (Shim-pack GLSS C18, 1.9 μm, 2.1*100, Shimadzu).

UPLC chromatographic conditions: mobile phase A is an aqueous solution containing 0.04% glacial acetic acid, and mobile phase B is a methanol solution containing 0.04% glacial acetic acid. Elution gradient: At 0 min, V_phase A_: V_phase B_ = 95:5; At 10 min, V_phase A_: V_phase B_ = 5:95; At 11 min, V_phase A_: V_phase B_ = 5:95; At 11.1 min, V_phase A_: V_phase B_ = 95:5, this ratio was continued until the end (duration is 14 minutes). The column temperature was set to 40°C, the injection volume of the injector was 2 μL, and the flow rate was 0.35 mL/min.

Orbitrap HRMS mass spectrometry conditions: ESI ionization method, mass spectrometry scanning mode is Full MS/ddMS2, ion collection mass range is 100~1200 m/z, and the lysis voltage is set to 20, 40, 60 eV; The spray voltage in positive ion mode is 3.5 kV, the capillary temperature is 350°C, the heater temperature is 350°C, the sheath gas (nitrogen) flow rate is 40 arb, and the auxiliary gas (nitrogen) flow rate is 10 arb. The spray voltage in negative ion mode is -3.0 kV, the capillary temperature is 350°C, the heater temperature is 350°C, the sheath flow rate is 30 arb, and the auxiliary air flow rate is 10 arb.

For wide-target detection of metabolites by UPLC-Q-Trap 6500+ multiplex reaction monitoring mode, the MRM detection window was set to 60 s, and the target cycle time was set to 0.8 s. The raw data were integrated by Multi Quant 3.0.3 to accurately obtain the relative content of each substance.

### 2.4 Metabolome analyses

All statistical analyses were carried out using R (4.1.1, http://www.r-project.org). (PCA) was performed using the R package “FactoMineR” (Lê et al., 2008) with 587 metabolite data from 3 biological replicates of mixed samples of each species to evaluate the metabolome differences across seven species. Circular plots were constructed using the R package Circlize, with the raw data normalized and scaled in the R program; the y-axes of the seven circles were set on the same scale. The hierarchical clustering tree using metabolome data of the seven species was constructed using the R package “hclust”. Orthogonal partial least squares discriminant analysis (OPLS-DA) was conducted by the R package “ropls” (Thévenot et al., 2015) to identify the major discriminant metabolite features among different species. A metabolite feature was considered a species-specific metabolic trait when matching the following criteria compared with the other six species: (i) *P* value of paired *t-test* ≤ 0.05; (ii) Fold Change ≥ 3. Venn diagrams were generated on the online website jvenn (http://jvenn.toulouse.inra.fr/app/index.html).

### 2.5 Enrichment analysis

Heatmaps were generated using the R package “pheatmap” with data normalization to divide the metabolome into eight clusters, and comparisons were performed via an average linkage method based on the Manhattan distance. Metabolic traits of different clusters were used for the Kyoto Encyclopedia of Genes and Genomes (KEGG) pathway enrichment analysis to elucidate the differential metabolic pathways among these seven different species. Enrichment analysis was carried out using the online platform MetaboAnalyst (https://www.metaboanalyst.ca/) selecting *Oryza sativa japonica* (Japanese rice) (KEGG) as a reference database. The bubble plot was drawn by the R package “ggplot2” (Ginestet, 2011).

## 3 Results

### 3.1 Metabolomic profiling of seven species

**Figure 1:**
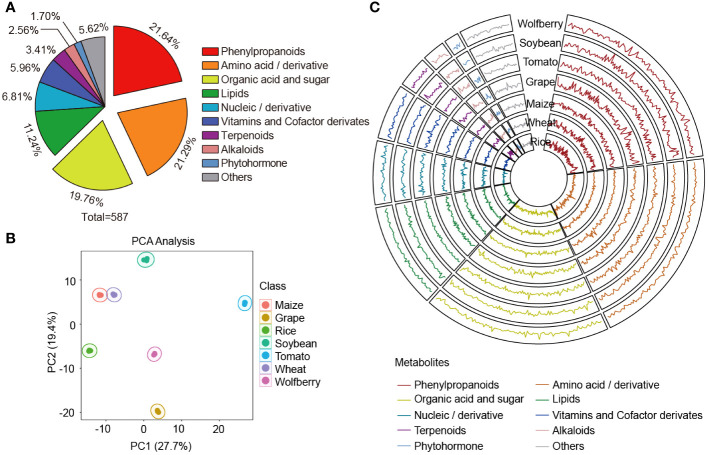
Multispecies metabolic spectrum composition analysis. **(A)** Classification of metabolites that have been speculated or verified. A total of 587 metabolites in wolfberry, soybean, tomato, grape, maize, wheat and rice were detected in this study. **(B)** Principal component analysis of the 587 metabolites among the seven species. **(C)** Features of the 587 metabolites in the seven species. Raw data were normalized and scaled using the R program, and data points show the average values of three replicates of each metabolite. The y-axes of the seven circles are on the same scale, from 0.508 to 1.556. Substance classes are represented by the line colors.

To dissect the metabolome differences between wolfberry and diverse species, six popular species were selected for comparison with wolfberry for analysis in this study. These species include four cereal crops maize, rice, wheat, legume crop soybean and two fruit crops tomato and grape. To reduce the specific error associated with a variety, we collected commercially available varieties of these seven species and mixed them to prepare mixed samples that represented the metabolome of each species. Collectively, a total of 1043 distinct metabolic features were detected and quantified in these seven species, the identification class is shown in **Supplementary Figure S1A**. Based on the comparison of our local metabolite database and commercial standards (Chen et al., 2014; Li et al., 2022b), 587 metabolites were identified, which could be divided into 10 categories, including 127 phenylpropanoids, 125 amino acids and their derivatives, 116 organic acids and sugars, 66 lipids, 40 nucleic acids and their derivatives, 35 vitamins and cofactor derivatives, 20 terpenoids, 15 alkaloids, 10 phytohormones and 33 other metabolites (**Figure 1A**, **Supplementary Table S1**).

Principal component analysis (PCA) was performed to assess the overall metabolome differences under the unsupervised model. The first principal component (PC1) explained the greatest variance (27.7%) of the variation among the seven species. Among the top 50 contributors of PC1, the proportion of amino acid and their derivative was the highest, followed by organic acids and sugars (**Supplementary Table S2**). The second principal component (PC2) was orthogonal to PC1 and was the largest in the remaining variance, accounting for 19.4% of the variance. Among the top 50 contributors in PC2, phenylpropanoids had the highest proportion, followed by amino acid and their derivative (**Supplementary Table S2**). For example, ornithine hydrochloride (ms025), the first-ranked identified metabolite, was found to accumulate more in the species with negative PC2 (grape, wolfberry and rice) than in those with positive PC2 (maize, wheat, soybean and tomato) (**Supplementary Figure S1B**). The results showed that the seven species were clearly separated, and three biological replicates of each species were compactly grouped together. This discrete clustering of the respective species indicates the distinct attributes of each species and the high repeatability and reliability of the experimental results (**Figure 1B**).

To visualize the variation in the metabolome among different species, we generated a circular plot for the seven species (**Figure 1C**). In the circular plot, strong variation in metabolic accumulation across seven species could be observed, with phenylpropanoids, nucleic acids and derivatives as well as phytohormones showing the most pronounced variation. To quantify the degree of variation, we used the coefficient of variation (CV) values of metabolites across the seven species. Phenylpropanoids were found to be the most variable metabolites, with a CV range from 11.56% to 244.93%, which is consistent with the results shown in the circular plot (**Supplementary Figure S1C**, **Supplementary Table S3**). In contrast, nucleic acids and derivatives have the least variation, probably because they are crucial components of genetic material in all species, some of which play vital roles in influencing the structure and function of RNA or in post-transcriptional gene regulation (Dominissini et al., 2016). Therefore, the conservation of these compounds, which represented the minimum variation in our data, implied their importance in living organisms, illustrating the role of nucleic acids and derivatives as building blocks in the life of organisms.

### 3.2 Differential accumulation patterns of metabolites among seven species

**Figure 2:**
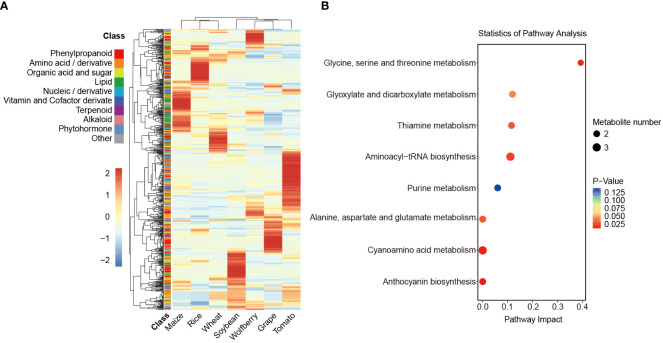
Analysis of differences in the relative content of metabolites. **(A)** Cluster analysis of the relative content of annotated metabolites in 7 species. The relative content of the annotated metabolites was represented by the mean of three biological replicates and normalized by z score standardization. Metabolites were clustered by hierarchical cluster analysis. Shades from blue to red in the figure represent increasing levels of metabolites, and the 10 color blocks in the class column indicate the classification of metabolites. **(B)** KEGG enrichment analysis of metabolites specifically accumulated in wolfberry. The *P* value ranges from 0.125 to 0.025, as seen by the color scheme of red to blue. The size of the dots represents the number of metabolites.

The overall profile of all identified metabolites in the seven species was analyzed by hierarchical cluster analysis (HCA). As shown in the heatmap, the metabolic diversity in different species was further indicated. Furthermore, all the compounds were hierarchically clustered into eight main clades, the first seven of which specifically accumulated in wolfberry, rice, maize, wheat, tomato, grape and soybean, indicating that each species has its preferential metabolites (**Figure 2A**, **Supplementary Table S4**).

To explore the preferred metabolic pathway of each species, we performed pathway enrichment analysis of the metabolites in clade 1 to clade 7 by the Kyoto Encyclopedia of Genes and Genomes (KEGG) database (**Figure 2B**, **Supplementary Figure S2**). Here, we focused on the preferred metabolite of wolfberry in clade 1. The 43 substances in clade 1 showed specific accumulation in wolfberry and were classified into 9 different categories. The two largest metabolites among them are organic acids and sugars and amino acids and their derivatives, accounting for 25.58% and 23.26%, respectively. Organic acids and sugars in wolfberry fruit are not only important nutrients but also the main influencing factors of flavor quality, while amino acids and derivatives are the main nutritional and medicinal components in LBFs (Zhao et al., 2020a). In the KEGG pathway enrichment analysis, the 43 metabolites were involved in 19 pathways. The major pathways are presented in the bubble plot (**Figure 2B**). In addition to amino acid metabolism, they also include glyoxylate and dicarboxylate metabolism, thiamine metabolism, aminoacyl-tRNA metabolism, purine metabolism, cyanoamino acid metabolism and anthocyanin biosynthesis. Similarly, 48 metabolites in clade 6 showed more accumulation in grapes and were enriched in the glutathione metabolic pathway. This could explain the antioxidant activity conferred by glutathione in grapes (**Supplementary Figure S2F**). Collectively, these results suggest that the specific accumulation of metabolites in different species can represent and determine their specific nutritional value.

### 3.3 Metabolic profile reflects the evolutionary relationship between monocotyledons and dicotyledons

**Figure 3:**
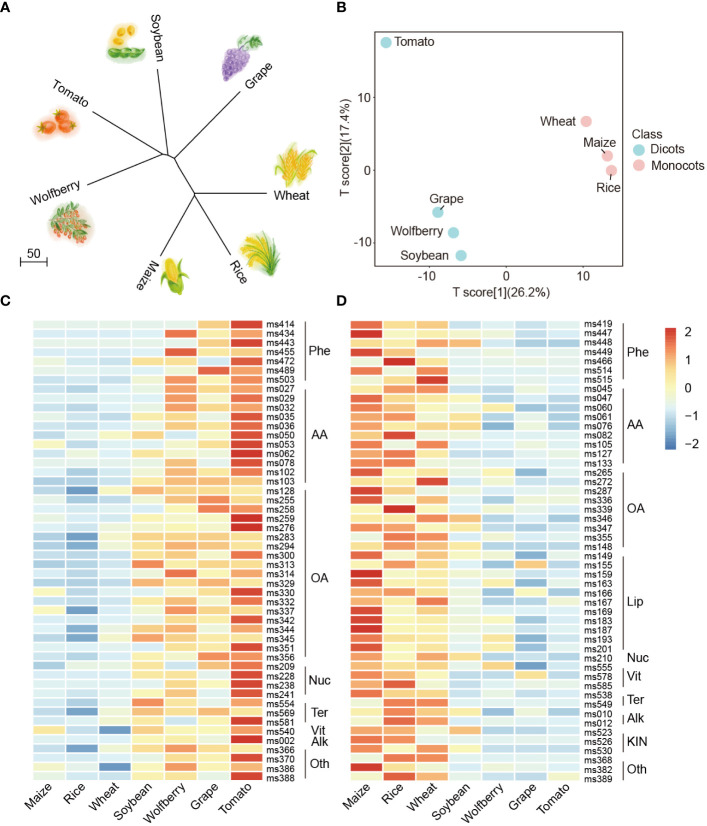
Metabolome analysis between monocotyledons and dicotyledons. **(A)** Neighbor-joining tree of the seven species with metabolome data. **(B)** PLS-DA analysis of the metabolite profiling of seven species. The data represent the mean values obtained from three biological replicates of each species. Powder blue and baby pink indicate dicots and monocots, respectively. **(C, D)** Comparisons of the relative accumulation levels of the top 50 **(C)** and bottom 50 **(D)** contributors identified in the PLS-DA distribution in the dicots and monocots. The relative content of metabolites was represented by the mean of three biological replicates and normalized by z score standardization. Metabolites were clustered by hierarchical cluster analysis. Shades from blue to red in the figure represent increasing levels of metabolites. Phe, Phenylpropanoids; AA, Amino acids/derivatives; OA, Organic acids and sugars; Nuc, Nucleics/derivatives; Ter, Terpenoids; Vit, Vitamins and Cofactor derivates; Alk, Alkaloids; Oth, Others; Lip, Lipids; KIN, Phytohormones.

To explore the affinities among the seven species, a neighbor-joining tree was constructed using metabolome data of the seven species (**Figure 3A**), and we also created a phylogenetic tree using the whole genome protein sequence of the seven species **Supplementary (Figure S3)**. The dicotyledons wolfberry, tomato, soybean and grape were clustered in both metabolome-based and protein-based trees, while the monocotyledons wheat, rice and maize were clustered together. These results indicate that there are differences in the metabolome between monocots and dicots, and the metabolomes could reflect the evolutionary relationship among different species.

To estimate the classification performance of monocots and dicots, we conducted supervised PCA and partial least-squares discriminant analysis (PLS-DA) among the seven species. The R^2^X, R^2^Y and Q^2^ are 0.436, 0.998 and 0.849, respectively, which are the prediction parameters of the PLS-DA model. The R^2^X value reflects the degree to which the model explains the variability of the input data, while the R^2^Y and Q^2^ values represent the model’s explanatory power for the output variable and its predictive accuracy, respectively. Here, R^2^Y and Q^2^ were both close to 0.9, with the same order of magnitude, indicating the stability and trustworthiness of this model (Golbraikh and Tropsha, 2002; Blasco et al., 2015; Su et al., 2022). The results showed that the main principal components (PC1 and PC2) explained 46.6% of the variability, with monocots and dicots being well discriminated from each other (**Figure 3B**).

The PLS-DA loading values of these 587 compounds are listed in **Supplementary Table S5** to quantify the contribution to the classification of the metabolites. On the basis of these data, two heatmaps were generated to organize the accumulation levels of the most effective contributors within monocots and dicots. We found that the top 50 contributors preferentially accumulated in dicots (**Figure 3C**, according to **Supplementary Table S5**, indicated in red), and organic acids and sugars (38%) as well as amino acids and derivatives (22%) were predominant among them, which might be identified as the specific accumulated components of dicots. In contrast, the bottom 50 contributors tended to have a preferential accumulation pattern in monocots (**Figure 3D**, **Supplementary Table S5**, indicated in blue), and it can be inferred that lipids and phytohormones accumulated specifically only in monocots. The aforementioned results reveal that the differentiation between monocots and dicots can mainly be ascribed to the considerable variation in compounds such as organic acids and sugars, amino acids and derivatives, phenylpropanoids and lipids. Together, these results indicate that the metabolome reflects evolutionary relationships between different crops.

### 3.4 Species-specific metabolites of the seven species

**Figure 4:**
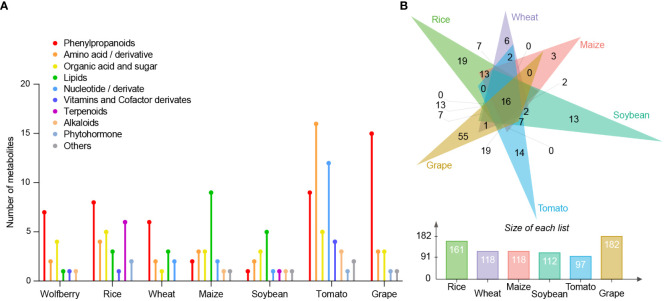
Comparison of differential metabolites between different species. **(A)** The number of known classified metabolites in each species showing more than threefold higher content than those in the other six species. The Y-axis refers to the number of metabolites. **(B)** Venn diagram analysis of wolfberry-specific metabolites relative to each species. Each color triangle denotes the wolfberry-specific metabolites relative to each species (top). Each color bar represents the number of wolfberry-specific metabolites relative to each species (bottom). The Y-axis represents the number of metabolites. Green: rice; purple: wheat; pink: maize; blue-green: soybean; blue: tomato; yellow: grape.

To identify those metabolites that could be used to split and distinguish the seven species, we calculated the fold change for each compound among the species. We defined the metabolites in a certain species whose content was more than 3 times higher than that of other species as species-specific metabolites. There were 16, 29, 14, 21, 15, 52 and 23 species-specific metabolites in wolfberry, rice, wheat, maize, soybean, tomato, and grape, respectively (**Figure 4A**). Seven phenylpropanoids (cyanidin chloride, echinacoside, esculoside, isorhamnetin 3-O-neohesperidoside, methyl p-coumarate, narcissoside and scopoletin), two amino acids and their derivatives (L-asparagine and N-acetylneuraminic acid), four organic acids and sugars (2-hydroxyisocaproic acid, 5-hydroxyhexanoic acid, phenyllactate and shikimic acid), one lipid (cholesterol), one vitamin and coenzyme derivative (riboflavin), and one alkaloid (4-amino-5-hydroxymethyl-2-methylpyrimidine) were among the 16 wolfberry-specific metabolites. Twenty-nine rice-specific metabolites comprised six terpenoids, two phytohormones, four amino acids and their derivatives, five organic acids and sugars, three lipids, eight phenylpropanoids and one vitamin and coenzyme derivative. Six phenylpropanoids, two amino acids and their derivatives, one organic acid and sugar, three lipids, and two nucleic acids and their derivatives are among the 14 wheat-specific metabolites. Among the 21 maize-specific metabolites were two phenylpropanoids, three amino acids and their derivatives, three organic acids and sugars, nine lipids, two nucleic acids and their derivatives, one alkaloid and one other compound. Fifteen soybean-specific metabolites included one phenylpropanoid, two amino acids and their derivatives, three organic acids and sugars, five lipids, one nucleic acid and its derivative, one terpenoid, one alkaloid and one other substance. Fifty-two tomato-specific metabolites included nine phenylpropanoids, 16 amino acids and their derivatives, five organic acids and sugars, 12 nucleic acids and their derivatives, four vitamins and coenzyme derivatives, three alkaloids, one phytohormone and two other compounds. In addition, 23 grape-specific metabolites were identified, comprising 15 phenylpropanoids, three amino acids and their derivatives, three organic acids and sugars, one phytohormone and one other compound.

We found that phenylpropanoids, organic acids and sugars accounted for the largest proportion of specific metabolites in wolfberry, which may determine the special nutritional value of wolfberry. To further compare the metabolites specifically accumulated in wolfberry compared to each species, we performed a Venn diagram analysis. The Venn diagram shows the number of wolfberry-specific metabolites compared with other species. Wolfberry had the most differential metabolites compared with grapes, followed by rice, corn and wheat, the three monocotyledon crops (**Figure 4B**). Venn diagrams of specific metabolites of other species are shown in **Supplementary Figure S4**. We found that all species had the highest number of specific metabolites relative to grape, while grape had the most specific metabolites compared to wheat.

### 3.5 Variation in the number of gene copies causes specific metabolites of Lycium barbarum to accumulate

**Figure 5:**
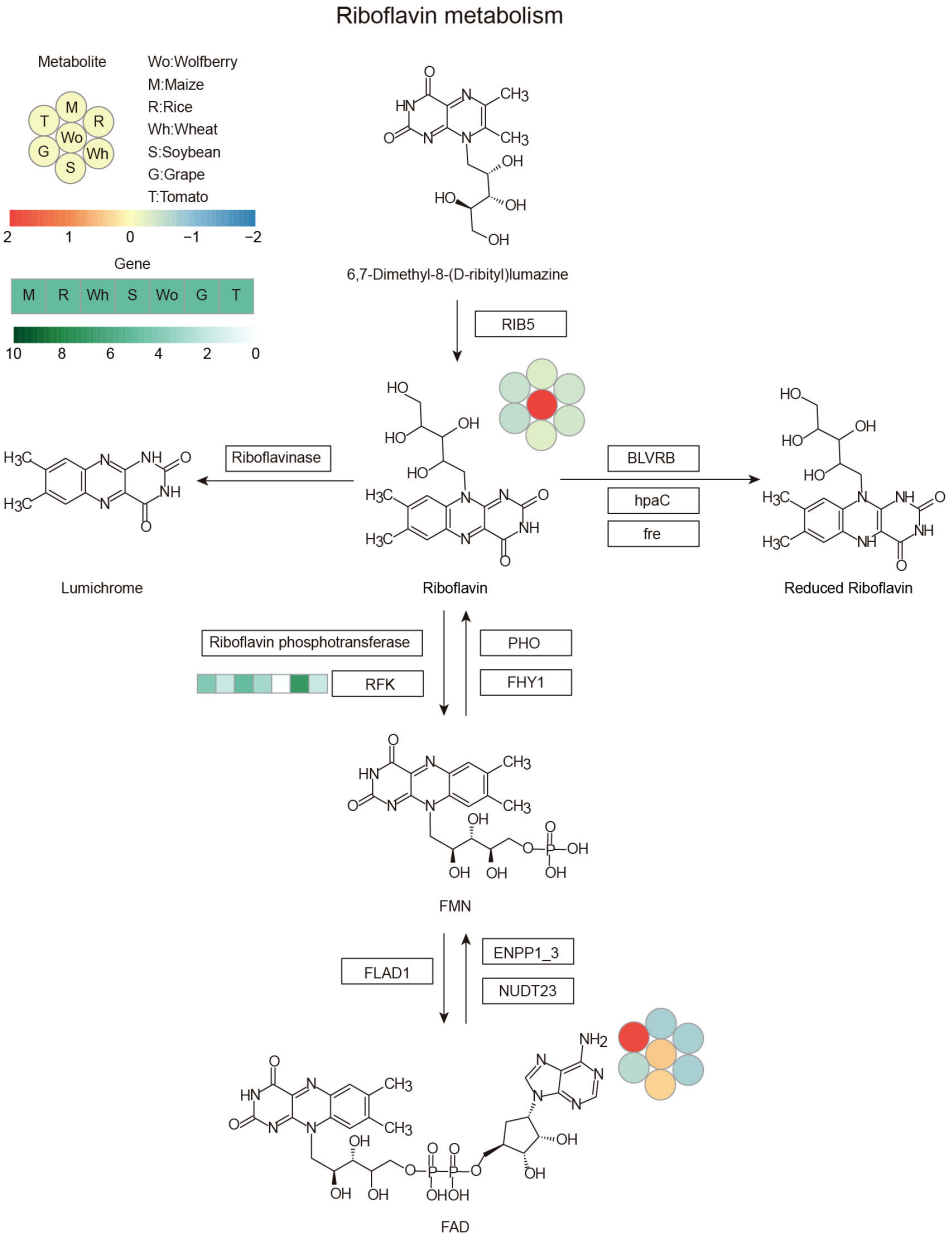
Riboflavin metabolic pathway. The heatmap shows the accumulation of metabolites and the copy number of genes among the seven species. Left corner there is a scale bar. The circles represent the accumulation levels of metabolites, and the data are represented by the average values of three biological replicates and normalized with z score standardization. Shades from blue to red represent increasing metabolite levels. The rectangular heatmap represents the copy numbers of genes, with green representing a high copy number and white representing a low copy number. The rectangle box indicates the key enzyme, and the abbreviation: RIB5, riboflavin synthase; BLVRB, biliverdin reductase/flavin reductase; hpaC, flavin reductase (NADH); fre, NAD(P)H-flavin reductase; PHO, acid phosphatase; FHY1, FMN hydrolase/5-amino-6-(5-phospho-D-ribitylamino) uracil phosphatase; RFK, riboflavin kinase; FLAD1, FAD synthetase; ENPP1_3, ectonucleotide pyrophosphatase/phosphodiesterase family member 1/3; NUDT23, ADP-ribose/FAD diphosphatase. Wo, M, R, Wh, S, G and T represent wolfberry, maize, rice, wheat, soybean, grape and tomato, respectively.

**Figure 6:**
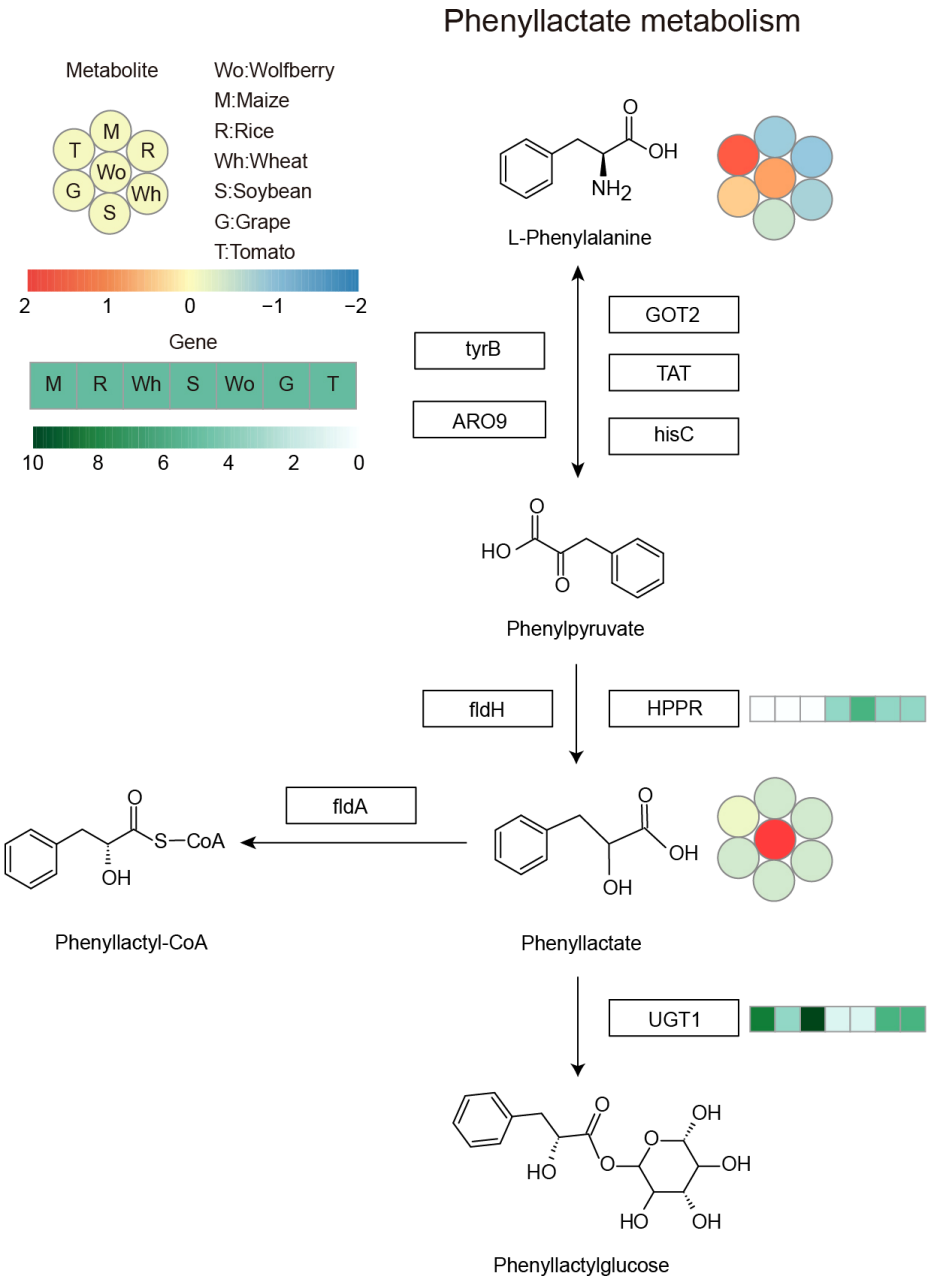
Phenyllactate metabolism pathway. The heatmap shows the accumulation of metabolites and the copy number of genes among the seven species. Left corner there is a scale bar. The circles represent the accumulation levels of metabolites, and the data are represented by the average values of three biological replicates and normalized with z score standardization. Shades from blue to red represent increasing metabolite levels. The rectangular heatmap represents the copy numbers of genes, with green representing a high copy number and white representing a low copy number. The rectangular box indicates the key enzyme, and the abbreviations are as follows: GOT2, aspartate aminotransferase, mitochondrial; TAT, tyrosine aminotransferase; hisC, histidinol-phosphate aminotransferase; tyrB, aromatic-amino-acid transaminase; ARO9, aromatic amino acid aminotransferase II; fldH, aromatic 2-oxoacid reductase; HPPR, hydroxyphenyl-pyruvate reductase; fldA, cinnamoyl-CoA:phenyllactate CoA-transferase; UGT1, phenyllactate UDP-glycosyltransferase. Wo, M, R, Wh, S, G and T represent wolfberry, maize, rice, wheat, soybean, grape and tomato, respectively.

The copy numbers of the riboflavin and phenyllactate degradation genes *riboflavin kinase* (*RFK*) and *phenyllactate UDP-glycosyltransferase* (*UGT1*) were lower in wolfberry than in other species, while the copy number of the phenyllactate synthesis gene *hydroxyphenyl-pyruvate reductase* (*HPPR*) were higher in wolfberry, suggesting that the copy number variation of these genes among species may be the main reason for the specific accumulation of riboflavin and phenyllactate in wolfberry. To illuminate the genetic basis of the high metabolite content in wolfberry, we investigated the metabolic pathway of riboflavin and phenyllactate, which are more than five times more abundant in wolfberry than in other species. *RFK*, encoding riboflavin kinase, which is responsible for the degradation of riboflavin, had the lowest number of copies in wolfberry (**Figure 5**). This could lead to the weakening of riboflavin’s ability to turn into flavin mononucleotide (FMN), thus increasing the accumulation of riboflavin in wolfberry. *HPPR*, encoding hydroxyphenyl-pyruvate reductase, which is responsible for synthesizing phenyllactate, has the highest copy number in wolfberry when compared with the other six species (**Figure 6**). As a result, the amount of phenyllactate in wolfberry may dramatically increase. Moreover, *UGT1*, encoding a key enzyme in the initial step of phenyllactate degradation, had the fewest copies in wolfberry (**Figure 6**). This will slow down the reaction of phenyllactate to phenyllactylglucose in wolfberry. Therefore, the increased synthesis and decreased degradation of phenyllactate are jointly responsible for the specific accumulation of phenyllactate in wolfberry (**Figure 6**). Taken together, our results showed that specific accumulation of riboflavin and phenyllactate in wolfberry may be caused by variation in the number of genes involved in riboflavin and phenyllactate synthesis or degradation.

## 4 Discussion

In this study, we discovered 16 metabolites that preferentially accumulate in wolfberry and showed that the copy number of genes may be one of the main factors contributing to the accumulation of these metabolites. We also demonstrate that metabolite contents can be utilized as indicators to determine the evolutionary relationship between various species.

In recent years, the nutritional components in plants have aroused broad concern. (Sun et al., 2024) Wolfberry has special nutritional value, and scholars have conducted extensive research on it. Over the past few years, with the advancement of metabolomic methods, metabolomic studies of various plants have been extensively studied, including wolfberry. Using widely targeted LC-MS/MS, the metabolites and their spatial distribution in dried kernels of six representative bread wheat cultivars in China were determined. Flavonoids varied the most in different varieties, and the concentration was higher in the outer layer of the grain but lower only in the kernel (Zhu et al., 2022). The metabolome study of maize and rice showed significant interspecific differences in their metabolic variation and identified flavonoids as the key constituent of interspecific metabolic divergence (Deng et al., 2020). A study with widely targeted metabolomics in three major food crops (wheat, maize and rice) and three fruits (banana, mango and grape) revealed that the main differential metabolites in crops and fruits were vitamins, amino acids, flavonoids and lipids and identified complementary patterns of essential nutrients in crops and fruits (Shi et al., 2022). Through the analysis of metabolites in wolfberry and tomato, it was found that the typical markers of tomato were lycopene, carotene, glutamate and GABA, while the characteristic metabolites of wolfberry were lycibarbarphenylpropanoids and zeaxanthin esters (Dumont et al., 2020). However, few direct comparisons have been made between the metabolomes of wolfberry and other common species. In this work, we demonstrated that the metabolomes of monocots and dicots differed significantly. By comparing wolfberry with other common species, we identified more undiscovered specific metabolites in wolfberry, which may be closely linked to wolfberry’s biological functions.

Numerous active ingredients have been identified in wolfberry. Lutein, zeaxanthin and carotene in wolfberry can reduce the risk of age-related macular degeneration (AMD) (Bertoldi et al., 2019). Together with vitamin B, they are responsible for the vision-improving effects of wolfberry (Kocyigit and Sanlier, 2017). Phenolic substances in wolfberry have been widely reported, and wolfberry has antioxidant, anti-inflammatory, neuroprotective, anticancer properties and intestinal microbiome regulatory effects (Ilić et al., 2020). In addition, LBPs, organic acids and carotenoids can also enhance the antioxidant capacity of wolfberry (Kocyigit and Sanlier, 2017). Changes in anthocyanin content are associated with the difference in fruit color of different wolfberry varieties and with the antioxidant capacity of the fruit (Zheng et al., 2011). In addition, wolfberry fruit also lowers blood sugar, blood fat and blood pressure, which is mainly due to the accumulation of amino acids, various monosaccharides and LBP in wolfberry (Potterat, 2010). In this study, we identified important active components in wolfberry. For example, betaine has been widely reported as an important active substance in wolfberry due to its antioxidant activity (Vidović et al., 2022). Our findings revealed that the concentration of betaine in wolfberry exceeds that of rice, maize, soybean, tomato, and grape by over 30 times, while its content in wheat is comparable to that in wolfberry (**Supplementary Table S2**). Besides, compared to those previously described in wolfberry, we discovered more metabolites that accumulate specifically in wolfberry, including N-acetylneuraminic acid, asparagine, 2-hydroxyisocaproic acid, 5-hydroxyhexanoic acid, phenyllactate, shikimic acid, echinacoside, esculin, isorhamnetin 3-O-neohesperidoside, narcissoside, scopoletin, cyanidin chloride, methyl p-coumarate, riboflavin, 4-amino-5-hydroxymethyl-2-methylpyrimidine and cholesterol. Of these, phenyllactate, N-acetylneuraminic acid, echinacoside, esculin, isorhamnetin 3-O-neohesperidoside, narcissoside and 4-amino-5-hydroxymethyl-2-methylpyrimidine were first identified as wolfberry-specific metabolites. Previous studies have shown that wolfberry can improve people’s vision (Kocyigit and Sanlier, 2017). Riboflavin, often known as vitamin B2, has been shown to sustain proper visual capabilities in living beings (Kocyigit and Sanlier, 2017). Wolfberry contains high levels of riboflavin, which suggests that riboflavin is responsible for the fruit’s ability to improve vision. Anthocyanins and organic acids in wolfberry have been reported to be associated with conferring antioxidant activity to wolfberry (Yan et al., 2018; Oğuz et al., 2021). In this study, we identified cyanidin chloride and four organic acids and sugars as wolfberry-specific metabolites, which may be significant anthocyanins and organic acids that confer antioxidant activity to wolfberry. Additionally, it has been reported that scopoletin has antioxidant activity in rats (Panda and Kar, 2006), and its specific accumulation in wolfberry may also be responsible for the antioxidant capacity of wolfberry.

The dissection of the genetic mechanism of important nutrient production in wolfberry is conducive to accelerating the process of plant breeding. Combining transcription and metabolism has become a widely used method to analyze the formation mechanisms of important metabolites in wolfberry. Using this research method, researchers have identified several key genes that regulate important metabolites of wolfberry through differences in transcription levels. For instance, the transcript level of *LbNCED1* positively regulates anthocyanin accumulation in wolfberry, thereby promoting fruit coloration. Nowadays, the genome has become a powerful tool for identifying functional genes (Zhao and Shi, 2022; Li et al., 2022a). The wolfberry reference genome is the first published reference genome of woody Solanaceae, which is beneficial for analyzing the genetic basis of wolfberry metabolites at the genome level. However, there is still a gap in the research analyzing the formation mechanism of wolfberry-specific metabolites at the genome level. In this work, we identified 16 wolfberry-specific metabolites, among which 10 exhibited a more distinct accumulation pattern in wolfberry. The concentration of these 10 metabolites in wolfberry was more than five times higher than in other species. Specifically, these metabolites include N-acetylneuraminic acid, 2-hydroxyisocaproic acid, 5-hydroxyhexanoic acid, phenyllactate, echinacoside, esculoside, isorhamnetin 3-O-neohesperidoside, narcissoside, scopoletin and riboflavin. Among them, the metabolic pathways of phenyllactate and riboflavin have been clearly analyzed in KEGG, we compared the copy number of the genes encoding key enzymes for the synthesis and degradation of specific metabolites in wolfberry and other species. Compared with other species, riboflavin and phenyllactate have more copies of key enzymes for synthesis and fewer copies of key enzymes for degradation in wolfberry, which may be the reason for their specific accumulation in wolfberry. Our work demonstrates that the formation of wolfberry-specific metabolites is controlled not only by the level of gene expression but also by the copy number of key genes that may lead to differences in metabolite synthesis pathways.

## 5 Conclusion

In this study, we compared the metabolome of wolfberry with that of six species, including the cereal crops maize, rice, wheat, legume crop soybean, and the fruit crops tomato and grape, and identified metabolites that accumulate specifically in wolfberry. Through high-throughput metabolomic analysis with widely targeted liquid chromatography-tandem mass spectrometry (LC-MS/MS), a total of 16 wolfberry-specific metabolites were identified, including seven phenylpropanoids, two amino acids and their derivatives, four organic acids and sugars, one lipid, one vitamin and coenzyme derivative, and one alkaloid. The phenyllactate degradation gene *UGT1* had the lowest copy number of the six species, whereas the riboflavin and phenyllactate synthesis genes *RFK* and *HPPR* had higher copy numbers than those of the other six species. This suggests that the copy numbers of *RFK*, *HPPR*, and *UGT1* may be the main reasons for the specific accumulation of riboflavin and phenyllactate in wolfberry. Moreover, the metabolome-based neighbor-joining tree showed that monocots and dicots clustered together separately, suggesting that metabolites could reflect the evolutionary relationship among different species. Taken together, we identified specific metabolites in wolfberry and provided new insight into the accumulation mechanism of species-specific metabolites at the genomic level.

## Data availability statement

The original contributions presented in the study are included in the article/**Supplementary Materials**. Further inquiries can be directed to the corresponding authors.

## Author contributions

QL: Data curation, Formal analysis, Writing – original draft, Writing – review & editing, Methodology. CZ: Writing – original draft, Methodology, Visualization, Writing – review & editing. HZ: Writing – original draft, Visualization. YZho: Writing – review & editing, Methodology. SL: Writing – review & editing, Visualization. YL: Writing – review & editing, Methodology. XM: Writing – review & editing. WA: Writing – review & editing, Resources. JuZ: Writing – review & editing. JiZ: Writing – review & editing, Funding acquisition. YZha: Writing – review & editing, Funding acquisition, Project administration, Supervision. CJ: Conceptualization, Funding acquisition, Project administration, Supervision, Writing – review & editing.
