# Supplementary material for: Comparative metabolomics combined with genome sequencing provides insights into novel wolfberry-specific metabolites and their formation mechanisms
Source: Front Plant Sci. 2024 Apr 26;15:1392175. doi: 10.3389/fpls.2024.1392175 (PMC11082402; doi:10.3389/fpls.2024.1392175)
Supplement: Supplementary file 1 [file DataSheet_1.docx]

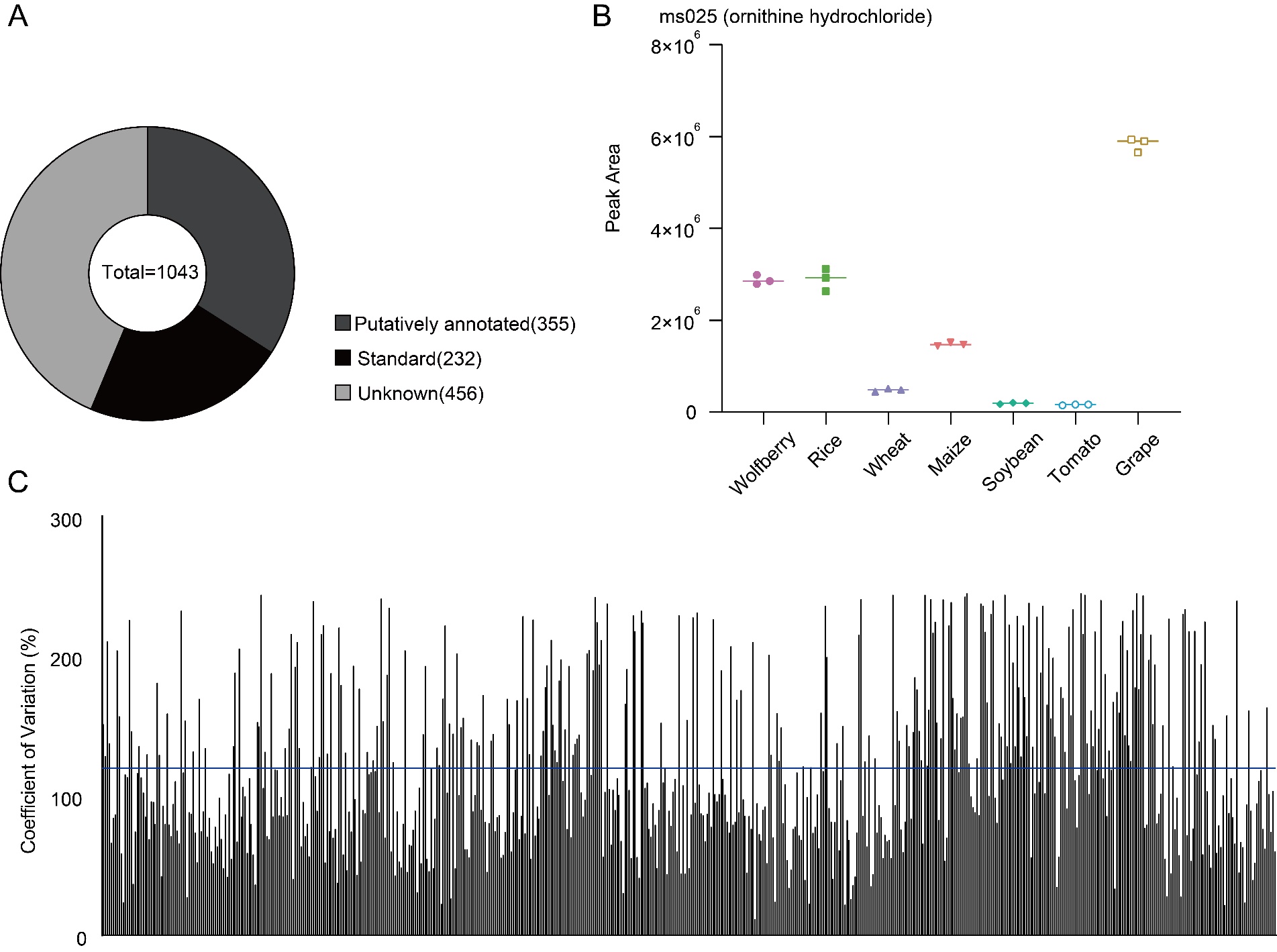


**Supplementary Figure 1** (A) Identification classes of detected metabolites obtained based on LC‒MS analysis and their numbers. (B) The relative accumulation of ms025 (ornithine hydrochloride) in these seven species. (C) Distribution of the coefficient of variation (CV) values of metabolic traits across the seven species.


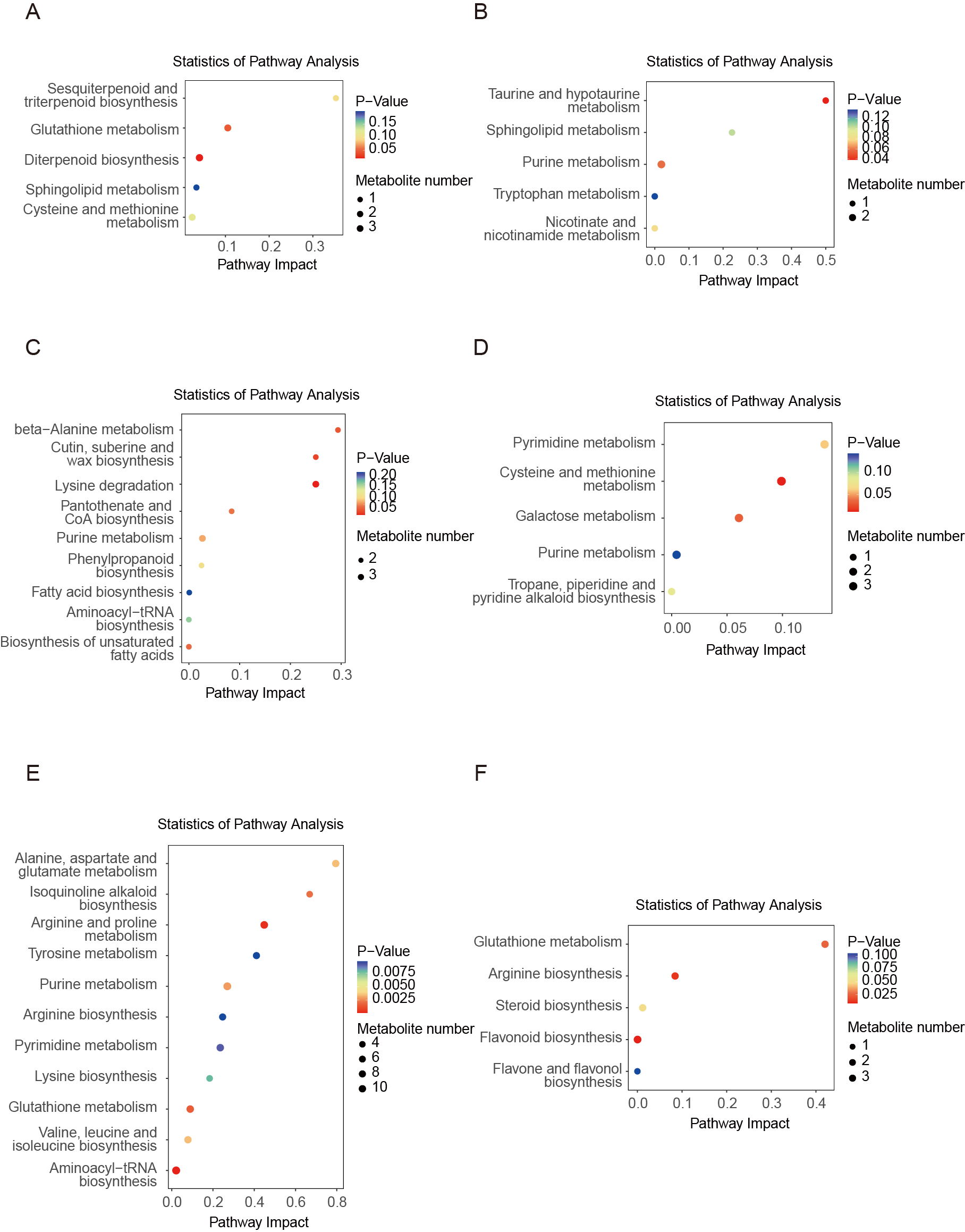


**Supplementary Figure 2** Pathway enrichment analysis of specific accumulated metabolites in (A) rice; (B) wheat; (C) maize; (D) soybean; (E) tomato; and (F) grape.


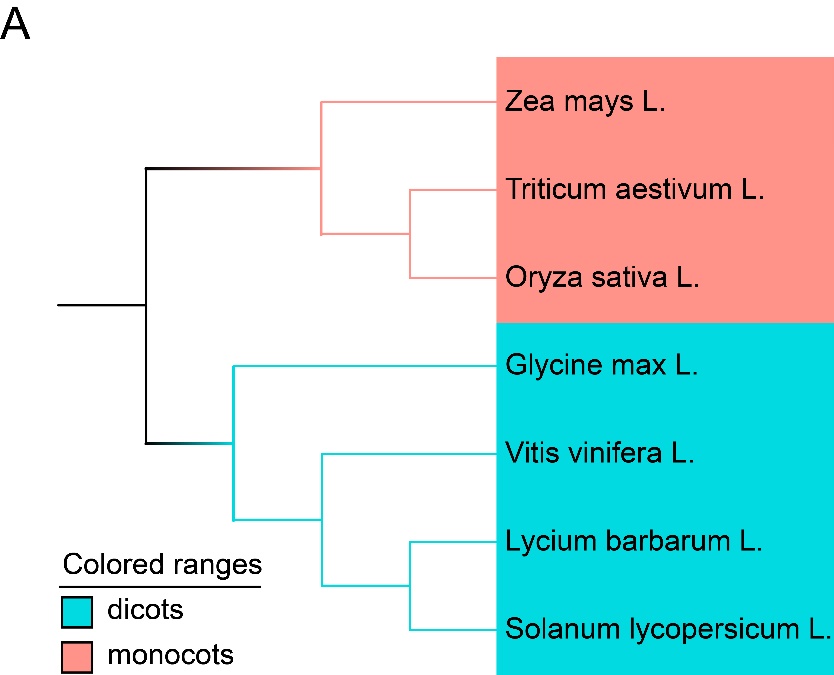


**Supplementary Figure 3** Phylogenomic analysis of seven species.

A phylogenetic tree was constructed using the single-copy protein data of the seven species.


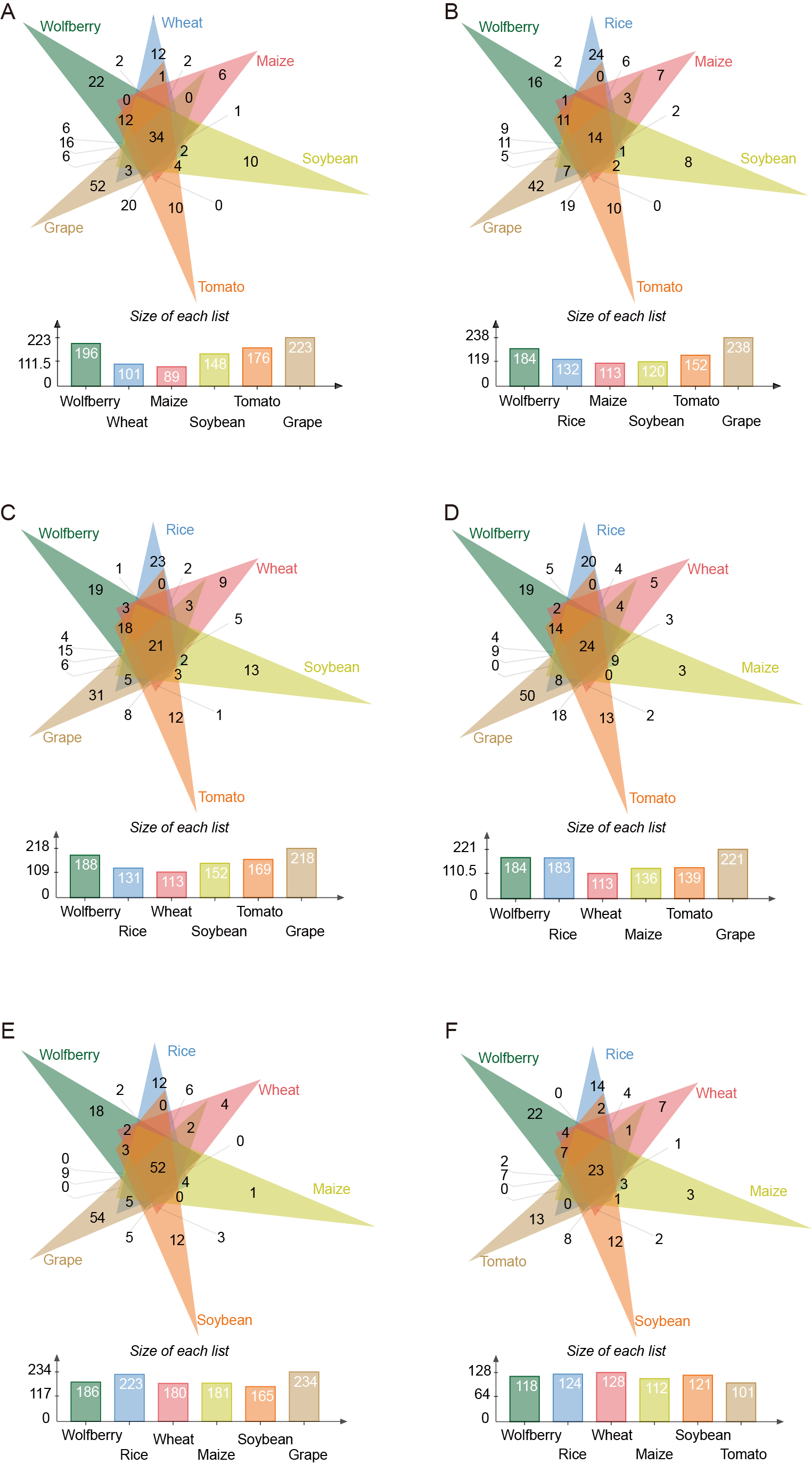


**Supplementary Figure** **4** Venn diagram analysis of specific accumulated metabolites of (A) rice; (B) wheat; (C) maize; (D) soybean; (E) tomato; and (F) grape.
